# Interest of neurofeedback training for cognitive performance and risk of brain disorders in the military context

**DOI:** 10.3389/fpsyg.2024.1412289

**Published:** 2024-12-13

**Authors:** Clémentine Jacques, Michael Quiquempoix, Fabien Sauvet, Michel Le Van Quyen, Danielle Gomez-Merino, Mounir Chennaoui

**Affiliations:** ^1^URP 7330 VIFASOM, Université Paris Cité, Paris, France; ^2^Unité Fatigue et Vigilance, Institut de Recherche Biomédicale des Armées (IRBA), Brétigny sur Orge, France; ^3^Inserm U1145, Université Sorbonne UMRCR2/UMR7371 CNRS, Paris, France; ^4^ThereSIS, THALES SIX GTS, Palaiseau, France

**Keywords:** neurofeedback training, military context, cognitive capacities, mental health, human performance

## Abstract

Operational environments are characterized by a range of psycho-physiological constraints that can degrade combatants’ performance and impact on their long-term health. Neurofeedback training (NFT), a non-invasive, safe and effective means of regulating brain activity, has been shown to be effective for mental disorders, as well as for cognitive and motor capacities and aiding sports performance in healthy individuals. Its value in helping soldiers in operational condition or suffering from post-traumatic stress (PTSD) is undeniable, but relatively unexplored. The aim of this narrative review is to show the applicability of NFT to enhance cognitive performance and to treat (or manage) PTSD symptoms in the military context. It provides an overview of NFT use cases before, during or after military operations, and in the treatment of soldiers suffering from PTSD. The position of NFT within the broad spectrum of performance enhancement techniques, as well as several key factors influencing the effectiveness of NFT are discussed. Finally, suggestions for the use of NFT in the military context (pre-training environments, and during and post-deployments to combat zones or field operations), future research directions, recommendations and caveats (e.g., on transfer to operational situations, inter-individual variability in responsiveness) are offered. This review is thus expected to draw clear perspectives for both researchers and armed forces regarding NFT for cognitive performance enhancement and PTSD treatment related to the military context.

## 1 The cognitive demands of the military in the operational environment

During sustained operations, deployments to combat zones, and basic training, the soldier, regardless of his service, Army - Air Force - Navy, may be exposed to a range of operational, environmental and physiological constraints likely to impair their operational capability, degrade their health and, ultimately, increase the risk of Posttraumatic stress disorder (PTSD). He/she may be exposed to extreme demanding operational tasks (physical and/or cognitive load using robotics, information technologies, artificial intelligence, for example) with high levels of anxiety and stress, and possibly severe environmental conditions with limited rest time to recover and limited sleep, and this with unpredictability (Trousselard et al., 2009; Sauvet et al., 2014; Good et al., 2020; Beckner et al., 2023). Operational deployments generally involve international travel, and Air force pilots or Navy submariners for example, may cross several time zones during the course of a single mission, which can also impact operational performance (Rabinowitz et al., 2009; Guo et al., 2020). Aircraft pilots in particular, and even Air Force « Drone » operators for several years now, are required to carry out multiple tasks, which can considerably increase their mental workload (Svensson et al., 1997; Bryant-Lees et al., 2021). For military personnel, the consequences of exposure to operational stresses go beyond a reduction in physical performance and translate into cognitive fatigue, which is subjectively associated with an increased sense of fatigue, reduced motivation, and a perceived lack of energy (Weeks et al., 2010; Miller et al., 2011; Main et al., 2023). Similarly, military units belonging to specific global intelligence organizations, such as the U.S. Air Force Distributed Common Ground Station (DGCS) which provides real-time situational awareness through the visual and technical intelligence needed to make strategic, operational, and tactical decisions on the battlefield. These units may be subject to higher levels of emotional exhaustion and psychological distress than those estimated for other military communities (Chappelle et al., 2019). This is how armies defined the concept of multi-domain operations: soldiers will operate at the nexus of a myriad of real-time data and information sources and face increasing pressure to multitask, prioritize, assess, decide, and act as opportunities and threats arise (U.S. Army Training and Doctrine Command, 2018; Herlihy, 2022). In addition, the weapons systems of the future will induce an excessively high cognitive load, requiring the cognitive abilities needed to manage several devices at once or to interact with brain-machine interfaces. Soldiers will have to process and adapt to large amounts of information, identify threats and respond to them, often in noise and with alterations of the normal wake/sleep cycle or sleep deprivation. It is therefore essential for military personnel to maintain effective cognitive performance in tasks that require sustained attention or concentration, as well as rapid information processing (Brunyé et al., 2020, 2022). In such a military context, leaders have a decisive impact on setting up and planning missions, promoting adequate preparation of combatants at strategic, operational, and tactical levels, improving sleep and fatigue management, and optimizing cognitive human potential on the battlefield (Billing et al., 2021; Teyhen et al., 2021).

The cognitive impairments described in laboratory scenarios of sustained military operations or during extended military training concern simple reaction time, vigilance, working memory, and reasoning (Lieberman et al., 2006, 2009; Vrijkotte et al., 2016; Beckner et al., 2021; Tait et al., 2024). Lieberman et al. (2006, 2009) demonstrated that cognitive function declined more extensively and rapidly than physical performance in U.S. Army officers from an elite unit during simulated combat with multiple stressors, with substantial degradation in visual vigilance, choice reaction time, and short-term memory, comparable to those in a field study conducted for an equivalent period of time in uncontrolled conditions. In addition, there is interindividual variability in the deterioration of cognitive functioning as a result of exposure to extreme environments which depends on the military task demands and the performance level (Paulus et al., 2009). The military tasks may involve storing, retaining, recalling, recognizing, and manipulating information, as well as planning, problem-solving or following goal-directed behavior. The degree of cognitive deterioration may also be affected by variables such as acclimatization to the environment, level of personal arousal and motivation (Martin et al., 2019).

Therefore, for several decades, a number of armed forces, most notably the U.S. Army, have been involved in research and development directly targeting cognitive performance enhancement, by seeking to improve attention and memory, situational awareness, decision-making and emotion regulation (Kelley et al., 2019; Brunyé et al., 2020, 2022). In the context of military operability, neuroenhancement is essentially pharmacological and involves the non-medical use of several stimulants, including caffeine, amphetamines and modafinil. Several of these show promise for temporary arousal and alertness, and can improve cognitive performance in soldiers under conditions of sleep restriction and deprivation (Brunyé et al., 2020). However, despite numerous publications and a high level of validation, the use of performance-enhancing drugs has been known to contribute to dependence, abuse, individual variability, and side effects (Marazziti et al., 2014). Thus, over the last decade, research teams have focused on studies regarding the effectiveness of neurofeedback training (NFT) to regulate brain activity and thereby to modify cognitive performance and behavior in healthy adults, and to help treat symptoms in PTSD patients (Gruzelier, 2014a; Navarro Gil et al., 2018; Yeh et al., 2021; Hong and Park, 2022; Uslu and Vögele, 2023). Neurofeedback training has been particularly studied for sports performance (Gong et al., 2021; Rydzik et al., 2023). The aim of this narrative review is to demonstrate the relevance of NFT for cognitive performance in the military context (pre-training environments, and during and post-deployments to combat zones or operations) and the related-PTSD risk.

## 2 Neurofeedback training for health and performance

### 2.1 NFT definition

Neurofeedback training (NFT) is a special case of brain-computer interface (BCI) providing real-time feedback on which individuals train to gain voluntary control of their brain activity through operant conditioning. Thus, individuals can learn to modify their brain activity through trial and error (Nowlis and Kamiya, 1970). This is a non-invasive, non-pharmacological method that records the electrical activity of an individual’s brain, measured by electroencephalography (EEG). It is an inexpensive and practical technique for training, conditioning and/or learning the self-regulation of brain waves (i.e., alpha, beta, alpha/theta, delta, gamma, and theta; Hammond, 2011). NFT is based on a closed-loop paradigm composed of four steps: (1) acquisition of neural data via EEG (typically 2 or 4 electrodes), (2) real time analysis of EEG signal to extract features of interest such as frequency or power from the frequency range (alpha, beta, delta, theta, and gamma) or a combination (a ratio) of these, (3) sensory feedback (on a visual, auditory or/and tactile modality) sent to the subject, depending on the fluctuation of the trained EEG parameter, (4) subject’s attempt to voluntarily take control of the sensory feedback (Sitaram et al., 2017). As an example, an interesting brain parameter (see section 2.2 for further discussion of the different brain parameters) is Sensory Motor Rhythm (SMR, ~12–15 Hz over the sensorimotor cortex), which is known to be related to states of calm and focused attention and has been used to improve shooting performance (Gong et al., 2020). A typical closed-loop paradigm would be an image whose contrast levels are modulated by SMR amplitude (steps 1–3), with higher contrasts displayed as SMR amplitude increases. The subject’s objective is to find a mental strategy (step 4) to maintain the higher contrast level, reflecting an increase in SMR amplitude (for more details on closed-loop methods: Bagdasaryan and Le Van Quyen, 2013; Marzbani et al., 2016; Enriquez-Geppert et al., 2017; Omejc et al., 2019).

Strategies can be very varied and depend on a number of factors. They obviously depend on the individual, but also on the EEG parameter to be neurofeedback-trained. For example, when training with SMR neurofeedback (frequency range 12 to 15 Hz), motor imagery can be suggested to the subject. Similarly, to decrease alpha waves (8–12 Hz), a state of relaxation is recommended, and conversely, to increase alpha waves, positive emotions are suggested.

By trying different strategies, a positive reinforcement or punishment stimulus is provided by the feedback and allows the subject to adapt (Sakurai and Song, 2016). For example, if the aim of EEG-neurofeedback is to increase the upper alpha amplitude and the subject adopts a strategy that allows him/her to have an amplitude above threshold, a positive reward is sent to the subject in the form of a pleasant sound (i.e., auditory feedback) or a clearer image (i.e., visual feedback). This reward can be delivered by different modalities, the most commonly used being visual, but also auditory and tactile as well as multimodal feedback (Gong et al., 2020; Omejc et al., 2019; Fleury et al., 2020). Conversely, if the subject does not find strategy, a punishment is sent in the form of an unpleasant sound, or visually (e.g., a square on a screen which turns red rather than green). Multimodal approaches have been shown to offer greater benefits than single-modality feedback, but the selection of an optimal modality remains a key topic of discussion in the literature, with specific modality choices adaptable to various brain parameters (Enriquez-Geppert et al., 2017; Vernon et al., 2003). Recent research efforts have focused on developing feedback modalities designed to enhance user motivation and improve learning outcomes beyond what is achievable with conventional 2D feedback. For example, multisensory animated scenarios (Cohen et al., 2016) and immersive virtual environments (Berger et al., 2022) have been shown to significantly facilitate learning processes.

It has also been suggested that learning ability in alpha neurofeedback training is predicted by resting alpha amplitude measured before training (Wan et al., 2014). In general, it has been suggested that NFT could be used to improve attentional processing through brain plasticity (Egner and Gruzelier, 2001; Ros et al., 2010; Davelaar et al., 2018). As the neural mechanisms underlying successful neurofeedback training still remain unexplained, Davelaar et al. (2018) demonstrated, through a theorical approach using computational methods from neuroscience, that it is precisely the striatal-thalamic and thalamo-cortical pathways that undergo synaptic modification governing the topographical and frequency specificity of learning. This theorical approach of neurofeedback learning also involves interoceptive homeostasis which may aid/reinforce learning though a probable interaction between the insula and the dopaminergic area of the midbrain.

However, despite the increasingly widespread use of neurofeedback technique, and although the choice of EEG frequency band and selection site for the EEG recording are well established, certain aspects of NFT methodology are not standardized, such as the feedback modality (auditory and/or visual), and the training conditions in terms of number of sessions, duration of a session or number of training blocks, and spread of sessions over time (Hammond, 2011; Marzbani et al., 2016; Mirifar et al., 2017). All these conditions also depend on the subject of interest, either for therapeutic purposes for defined pathologies, or for athlete or military performance. What’s more, whatever the success of NFT, in many cases its effectiveness often varies according to the individual, with a significant proportion of subjects being unable to control brain metrics even after multiple training sessions (responder/non-responder notion). These subjects are generally considered “non-learners,” as opposed to “learners” (Alkoby et al., 2018). So, in 2020, neurofeedback researchers proposed a consensus-derived checklist that aims to improve reporting and experimental design standards in the field (Ros et al., 2020).

### 2.2 Areas of NFT application

Kamiya’s NFT experiment in the late 1960s, which first described a person’s ability to learn to control the electrical activities of the brain by voluntarily increasing the amplitude of EEG alpha band oscillations (8–12 Hz), revealed a positive psychophysiological impact as subjects reported states of relaxation and pleasant feelings (Kamiya, 1969). Thereafter, several studies developed the connection between alpha oscillation and cognitive processes, notably attention and inhibitory control and timing (Klimesch, 1999, 2012; Klimesch et al., 2007). At the same time, the use of different NFT protocols (sensory motor rhythm, SMR; alpha amplitude; alpha/theta ratio) has been shown to reduce stress and anxiety in healthcare professionals such as ophthalmic surgeons practicing surgical techniques in simulation conditions (Ros et al., 2009), as well to improve the performance of sports and arts professionals (Gruzelier, 2014a, 2014b, 2014c). In this sense, a single session of SMR NFT has been shown to benefit anxiety (using psychometric measures of mood) and salivary cortisol levels (Gadea et al., 2020). Regarding NFT based on EEG alpha activity, Ossadtchi et al. (2017) demonstrated that the alpha power rose because of an increase in the incidence rate of alpha episodes, whereas the amplitude and the duration of alpha oscillations remained unchanged. These authors called for more investigation into discrete EEG features as an approach to improve neurofeedback efficiency and explore and quantify the neurofeedback-induced effects.

Some previous data in the literature have suggested that NF could be a promising alternative or complementary therapeutic option to stimulant treatment for the symptoms of Attention Deficit Hyperactivity Disorder (ADHD), but this still remains a matter of debate (Riesco-Matías et al., 2021; Chiu et al., 2022; Rahmani et al., 2022). In a recent meta-analysis, Chiu et al. (2022) indicated that surface electroencephalographic neurofeedback has beneficial effect on sustained attention in ADHD, with limited effect on selective attention, and superior effect of beta wave enhancement than of theta/beta ratio reduction or modulation of slow cortical potential. On the basis of some published work showing the effectiveness of NFT in stabilizing or even increasing attentional performance in ADHD patients (Deiber et al., 2020; Chiu et al., 2022), the idea has been floated of using it to improve peak performance in healthy subjects.

Behavioral positive effects of NFT (later called “transfer effect,” i.e., maximum transfer of information that increases neuronal efficiency) in healthy subjects have been observed on vigilant attention and sustained attention with decreased reaction times on the Psychomotor Vigilance Task (PVT; through fNIRS-NFT; Pamplona et al., 2020) and on the Stroop’s test (through EEG-NFT on alpha power magnitude; Berger and Davelaar, 2018). Not only response speed (reaction times) can be enhanced, but also the accuracy of information processing and executive control components of attentional capacities, as it has been shown at the Continuous Performance Task (CPT) measuring visual attention (through EEG-NFT on SMR activity; Vernon et al., 2003) and at the Attention Network Test (ANT) measuring the three attention networks, the alerting, orienting and executive components (through EEG-NFT on frontal-midline theta activity; Wang and Hsieh, 2013). Relative to NFT based on EEG alpha neuromodulation, studies evidenced positive effects of training on upper alpha frequency amplitude for several cognitive capacities (Zoefel et al., 2011; Enriquez-Geppert et al., 2017; Li et al., 2023). After five sessions of NFT over 1 week, Zoefel et al. (2011) showed enhanced performance in two tests of mental rotation performance which illustrated different stages of spatial thinking [the perceptual stages (perceptual processing, identification and discrimination of stimuli, identification of orientation)], the rotation process itself (mental rotation, judgement of parity), and decision processing stages (response selection, execution). A single session of individual upper alpha enhancement (25 min) improved performance in a mental rotation task, as it did in sham-controlled subjects, but the improvement was more marked for the NF group (Escolano et al., 2011). In a recent study, Li et al. (2023) showed improved performance in a mental rotation test and the N-Back working memory test after 2 days of NFT based on Individual Alpha Frequency (IAF) in both the neurofeedback and sham groups, however, the improvement in the neurofeedback group was more pronounced and correlated with the upregulation of IAFs. Even more recently, a long period (4 weeks) of auditory alpha NFT successfully induced alpha wave and improved short-term memory of healthy subjects (Takabatake et al., 2021).

In sports training, unlike traditional training which aims to strengthen endurance and speed, NFT mainly focuses on the mental state. Thus, a properly planned and carried out NFT by athletes has been shown to improve many variables and their sports performance (reduction in stress and anxiety levels, increase in the ability to self-control physiological factors, improvement in behavioral efficiency and improvement in reaction time and decision-making; Mirifar et al., 2017; Brito et al., 2022; Rydzik et al., 2023). The various athletes concerned were soccer goalkeepers, archers, golfers for visual and spatial attention performances, elite swimmers for anxiety, etc. In 2020, Gong et al. evidenced a significant improvement in the shooting performance after SMR NFT (6 sessions of 25 min over 3 weeks) whereas there was a decrease after EEG alpha NFT in non-professional shooters. Thereafter, from the perspective of user experience, the same author summarizes NFT’s concept, process, and research methods and puts forward an SP–NFT classification method for improving sports performance (Gong et al., 2021).

### 2.3 NFT vs. other technologies

**Figure 1:**
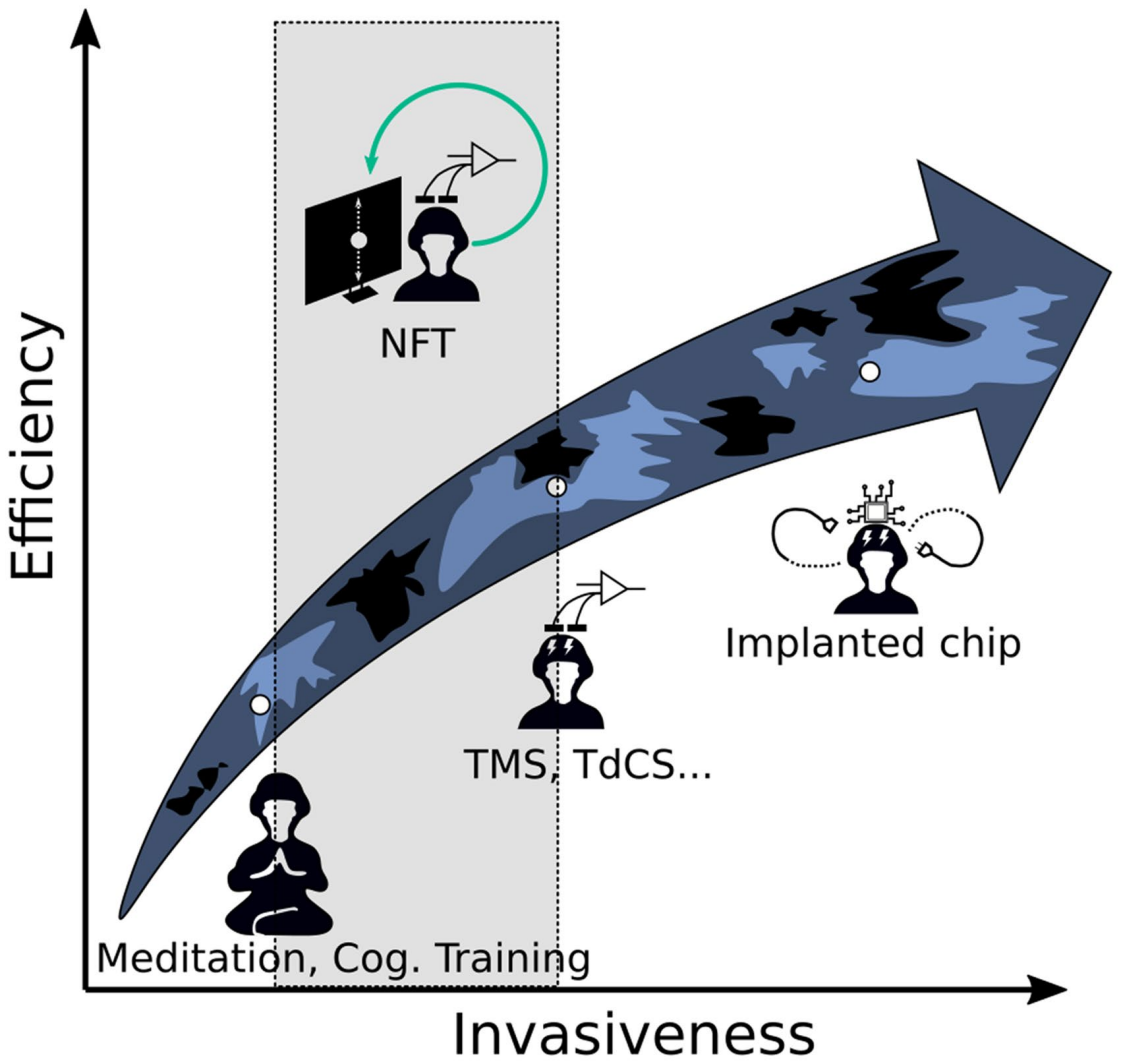
Efficiency-invasiveness relationship of different brain training technics. The efficacy-invasiveness relationship for several cognitive training technologies and methods described in the literature.

Achieving the best athletic/military operational performances depends not only on highest physical fitness level (strength, endurance) and the corresponding specific training, but also on the improvement of the speed and accuracy of decision-making abilities. Research into cognitive enhancement for military purposes is not new, and numerous studies have explored different approaches, from the more invasive, using psychoactive stimulants for example, to non-invasive brain stimulation (NIBS) systems, including transcranial direct current stimulation (tDCS) and transcranial magnetic stimulation (TMS), to the more natural methods of self-improvement, such as meditation or cardiac coherence (Kelley et al., 2019; Figure 1).

In contrast, only a few studies, cited later in this review, have investigated the interest of NFT for improving cognitive performance in the military operational context. NFT is based on brain monitoring devices, enabling users to receive (sensory) feedback on their own neural activities in real-time. These devices are very minimally invasive, of the same order as NIBS, and halfway with natural self-improvement techniques.

As with NIBS, whose use was primarily for therapeutic purposes aimed at restoring impaired brain function, there is a growing body of scientific literature on the use of NFT for self-improvement and performance. Nevertheless, there are crucial differences between NFT and NIBS. Firstly, the main difference lies in the involvement of subjects in the use of the technique. NIBS relies on passive brain stimulation to alter neural firing (TMS) or excitability (TES, tDCS). Although NIBS are effortless and painless and have an exciting potential role in modulating brain activities to increase cognitive function in a military context, consent, and acceptance to receive magnetic or electrical stimulation remains a major ethical issue (Feltman et al., 2020). On the contrary, NFT requires participants’ voluntary effort to control their own brain activity by interacting with playful video or audio games. This offers an important advantage over NIBS by avoiding problems of consent, as the user’s agency is necessary to effectively practice NFT. Secondly, NIBS are expensive (especially TMS), currently non-portable and require expertise, and the question of transferability of favorable cognitive effects from the lab to real-world tasks is still open (Feltman et al., 2020). In contrast, NFT devices based on EEG and near-infrared spectroscopy (NIRS) have a great portability for use in the field or at home, and competitively priced for potential mass distribution to armed forces.

A plausible rationale of the added value of NFT over natural self-improvement methods (meditation), is the lack of feedback and meta-feedback during the learning process of the latter. Indeed, the NFT principle relies on the occurrence of real-time feedback, reflecting the on-going brain signal fluctuations and by extension the user’s instantaneous performance in his or her ability to voluntarily modulate this neural activity. In addition, meta-feedback are given throughout a NFT session, divided into short blocks of active training, informing about user’s progression. At the opposite of meditation-like technics, these features of NFT make it possible to objectify and accelerate the learning process of voluntary modulation of neural activity. As a result, in recent years, there has been growing interest in the use of NFT to increase cognitive capacities, primarily in the field of expert sports performance, but also for military operational readiness.

## 3 NFT: interest for the military performance and health

### 3.1 Interest for performance in the operational environment

Three non-exclusive objectives are sought when considering a person’s ability to achieve his or her goal, whether in sporting competitions or military operations: maintain/improve performance, strengthen resilience in the face of adversity, and optimize recovery of cognitive processes. As mentioned in the previous sections, there are many cognitive functions trainable with neurofeedback which are of interest insofar as they are limiting factors for performing a huge number of complex (military) tasks, including attentional capacities, inhibition, working memory, emotion regulation and stress/anxiety management (Blacker et al., 2019). The literature is fairly broad in considering that NFT is beneficial for several components of attentional function (Gruzelier, 2014a, 2014b, 2014c), while there is no certainty about efficacy on executive function abilities (Gordon et al., 2020; Viviani and Vallesi, 2021).

In recent years, we have seen the emergence in the military context not only of a human-human team, but also of human-machine cooperation, which has rapidly evolved into a new mode of combat (Stowers et al., 2021). Such a hybrid team provides a combination of human decision making and machine information sharing chain, with a high degree of cognitive demand. Of note, cooperative behaviors between several persons to achieve shared objectives are correlated with a neurophysiological phenomenon described as inter-brain synchrony (IBS). IBS reflects a higher coherence between brain electrical activities in a specific frequency range and is thought to underlie higher group performance during a complex task such as landing an aircraft or in-flight refueling (Toppi et al., 2016). In a recent review, Lu et al. (2022) evidenced beneficial effects of transcranial electrical stimulation for military pilots to improve their teamwork. To date, no studies using NFT have been conducted on group performance, although it has been shown that IBS can be achieved with neurofeedback (Müller et al., 2021). These converging results make NFT a very promising tool for military skills requiring a higher level of cognitive functioning. When it comes to any type of training (cognitive in this case), the notion of transfer to performance in more ecological (or operational) situations is crucial and still the subject of debate (Blacker et al., 2019), although there is a growing literature on the transfer of NFT to sports performance (Mirifar et al., 2017; Rydzik et al., 2023), or to ecological situations such as driving (Balconi et al., 2019).

**Figure 2:**
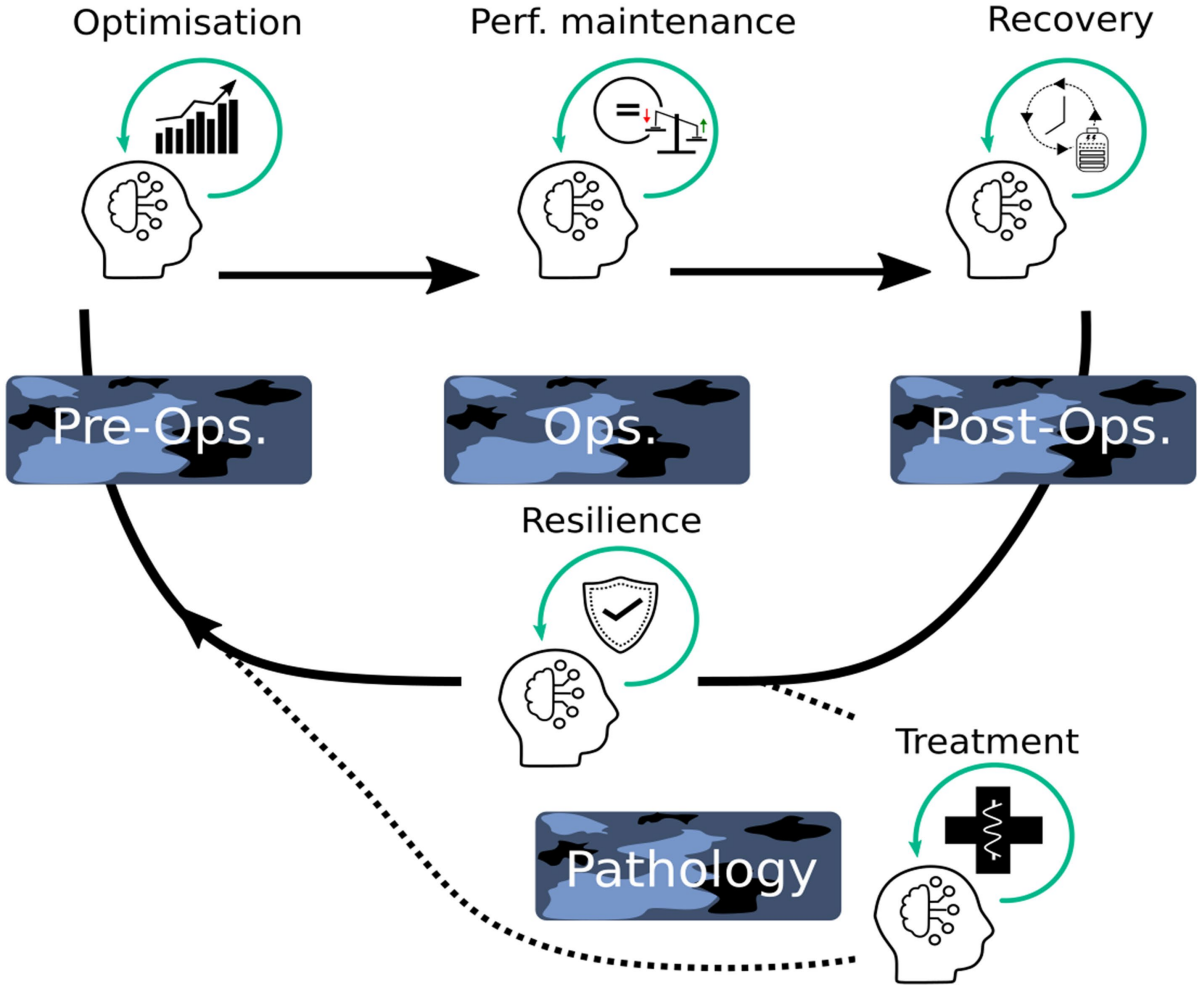
Neurofeedback training use-cases and intended effects as a function of operational context. Ops: military operation. Neurofeedback training use cases and expected effects according to the military operational (Ops) context (i.e., pre-, during, and post-).

To our knowledge, there are no studies on the effects of NFT on soldiers’ cognitive performance for military-specific task performance or during operational settings, although it is a technique they could theoretically use at various stages of their military activities to improve operational efficiency. The operational settings include, but are not limited to, training environments, field operations, and deployment to combat zones (Kelley et al., 2019). Thus, the aim of this section is to propose a conceptual framework for the use of NFT in the Armed forces (Figure 2).

The simplest use case to consider for NFT might be in the operational training phase of soldiers, including the training of young recruits, bearing in mind the notion of motor performance. A recent meta-analysis indicated that there is a dose–response gradient between NFT and improved motor performance, and that NFT of more than 1 week and more than 125 min of cumulative training time would be recommended to improve motor performance (Onagawa et al., 2023). Thus, one study reported that an SMR NFT protocol, fifteen 60-min sessions over the course of 5 weeks with three sessions per week, improves rifle shooters’ performances (Rostami et al., 2012). Another study also reported enhancement of the level of attention after 20 NFT sessions oriented towards strengthening the beta frequency, in students at the Military University of Technology, who are professional soldiers (Mikicin et al., 2018). When it comes to preparing soldiers, this phase is also conducive to the development of resilience, i.e., a person’s ability to maintain integrity and performance in face of adversity. Thus, in a recent study, Keynan et al. (2019) developed an NFT program modulating amygdala activity and aimed at improving stress regulation in a large cohort of healthy soldiers undergoing stressful military training. They found greater emotional recognition and better performances at the emotional Stroop, which indicates that NFT participants were more able to deal with stress relative to control non-NF. In a follow-up fMRI (functional magnetic resonance imaging)-NF, they also evidenced greater amygdala blood-oxygen-level-dependent downregulation and amygdala–ventromedial prefrontal cortex functional connectivity following the amygdala neurofeedback training relative to control non-NF. This study paves the way for the use of NFT to improve soldier resilience, as its findings suggest that trained soldiers faced with adverse events during operations will cope better with stress, limiting the decline in their operational performance on the battlefield. Finally, these authors suggest that their NFT of the amygdala could prevent soldiers from developing trauma-related psychopathologies, in the hope of reducing the incidence of PTSD. This has been followed by a proof-of-concept NFT study aimed at down-regulating limbic activity in non-military PTSD patients, leading to immediate clinical improvement (Fruchtman-Steinbok et al., 2021).

When considering the training phase, it is obviously necessary to achieve the highest, most robust operational performance at the end of this phase, but also to optimize it, as this is a crucial step in guaranteeing the availability of prepared soldiers in a context of potentially low renewal capacity. In this respect, it is worth introducing NFT to improve attention, working memory as well as stress regulation, in order to speed up the process of learning military skills. A more tricky use of neurofeedback principle (i.e., self-control of brain oscillations based on a sensory feedback) could be concomitantly with an ongoing specific military task to increase and/or to maintain task-related performances. It has thus been demonstrated that an NFT intervention during concomitant physical training sessions in swimmers can optimize psychomotor activities (Mikicin et al., 2020). The pioneering work of Faller and colleagues demonstrated the feasibility of a real-time modulation of arousal via neurofeedback during a simulated aerial navigation (boundary avoidance task) in virtual reality (Faller et al., 2019). After identifying EEG correlates of stress-related over-activation of anterior cingulate cortex (ACC), which is associated with hyper-arousal and poorer performances, the authors asked healthy subjects to moderate ACC activity (via an auditory feedback) when it exceeds a threshold during the boundary avoidance task. They found an increase in performance (more time spent on flight) for subjects who managed to voluntarily modulate ACC activity while doing the task, suggesting that (i) self-control of brain activities is feasible simultaneously with a cognitively-demanding task, (ii) the dual task (neurofeedback + boundary avoidance task) does not decrease behavioral performances and (iii) this strategy should be further explored with more ecological situations or operational tasks. In a recent study, a cognitive training coupled with neurofeedback using NIRS (CT-NF) led to increased activity in the dorsolateral prefrontal cortex and has higher beneficial effects on episodic memory, working memory and attention compared to cognitive training salone (CT) and active control (ACT; Nouchi et al., 2021). A few years ago, Blacker et al. (2019) reported that neurofeedback combined with cognitive training could boost performance in a number of military-relevant scenarios (Blacker et al., 2019).

At the opposite of hyper-arousal, one of the main constraint faced by military in operation is mental fatigue, mainly due to a poor sleep quality/quantity leading to sleep debt (sleepiness). A previous study of our lab detected low-vigilance states during real flights in military French pilots, using a miniaturized single EEG channel recording device associated to an automatic algorithm (Sauvet et al., 2014). Interestingly, a preliminary study has designed a NFT based on the modulation of (alpha + theta)/beta ratio that can reduce daytime drowsiness (Monseigne et al., 2019). Another approach could be to improve and optimize soldiers’ sleep, thus reducing risks of sleepiness during their operations. NFT interventions to enhance sleep of healthy participants revealed very few benefits (mainly on sleep spindles quantity and subsequent memory consolidation capacities; Berner et al., 2006; Hoedlmoser et al., 2008), suggesting little to no impact of NFT on normal sleep. However, the extensive literature on the positive effects of NFT on sleep parameters in patients with insomnia or ADHD (Hammer et al., 2011; Arns and Kenemans, 2014) leaves room for possible improvements in degraded sleep situations, with further work on the use of NFT to improve soldiers’ sleep in operational environments needed.

Finally, in the military context, NFT might be not only used before (training environments) and during military operations but should also be considered after a mission for recovery purposes. In this respect, NFT targeting stress after a military operation seems particularly interesting because of its ability to improve emotion regulation (Keynan et al., 2019) and to decrease anxiety and cortisol levels (Gadea et al., 2020).

Although NFB can be performed using fMRI or fNIRS, EEG-based systems are clearly most suitable for operational use in the field. Consumer-grade EEG wearables are becoming more and more numerous and of better quality, with the advantage of requiring little expertise thanks to the advent of dry electrodes. EEG recordings can be made with laptops or even smartphones, leaving great opportunities to provide real-time feedback outside of the laboratory. Some work has provided interesting information on the combination of EEG-NFB and the use of Virtual Reality (VR) headsets (Faller et al., 2019; Berger and Davelaar, 2018; Berger et al., 2022), increasing the portability and immersive capabilities of neurofeedback training. However, given the practical aspects of NFB training, further work is needed to better specify the NFB protocols to follow, in terms of intensity, duration and number of training sessions, in based on desired outcomes (whether optimal performance or symptom improvement).

### 3.2 Interest in the treatment of pathologies-related to the military context

Numerous studies have shown that the stressful environment surrounding the military, mainly that of combat operations, is negatively associated with the performance of relevant military skills, and increased risk of brain disorders. Posttraumatic stress disorder (PTSD), mild traumatic brain injury (mTBI), chronic pain, and the associated sleep disturbances, are highly prevalent in military personnel and veterans (Moore et al., 2020; Oberman et al., 2020; Saguin et al., 2021). In the military operational context, the increased risk of TBI can be due to falls, car accidents or violent impacts, and can be caused by shock waves induced by explosive weapons, including improvised explosive devices (IED) and heavy munitions firing, the majority of TBI being classified as mild (mTBI; Kim et al., 2023). All these brain disorders linked to military operations can have a negative impact on military performance. Indeed, people with mTBI often report acute symptoms such as dizziness, nausea, difficulty sleeping, diminished attention, amnesia, or headaches, as well as other cognitive deficits such as memory acquisition, slowed processing speed, multitasking problems, loss of train of thought and overall cognitive functioning (Oberman et al., 2020). On the other hand, PTSD, a debilitating psychiatric disorder that can develop after exposure to trauma, involves alterations in cognition and mood (negative beliefs and expectations, difficulty concentrating and inability to experience positive emotions), as well as alterations in arousal and reactivity (aggression, destructive behaviour, hypervigilance, and sleep disturbances; American Psychiatric Association, 2013).

Several studies have shown that NFT can be useful for patients with PTSD (Gray, 2017; Nicholson et al., 2020; Hong and Park, 2022; Askovic et al., 2023) and TBI (Reiter et al., 2016), resulting in symptom improvement and, when studied, normalizing aberrant neural dynamics, but results specifically for military sufferers are limited. With respect to brain activity among persons with PTSD, alpha-rhythm reductions have been associated with PTSD symptoms, particularly those of chronic hyperarousal (Ros et al., 2014; Nicholson et al., 2020). In a preliminary double-blind sham-controlled randomized trial, Nicholson et al. (2020) demonstrated for the first time that EEG-NFB based on the alpha-rhythm over 20 weeks led to reductions on PTSD severity scores as well as increased rates of PTSD remission. Furthermore, they showed that the aberrant patterns of default-mode network (DMN) and salient networks (SN) connectivity detected in PTSD patients at baseline (compared to healthy controls) tended to normalize after NFB treatment. In a second study, Nicholson et al. (2023) confirmed these preliminary findings and further showed that at the three-month follow-up assessment, 60.0% of participants in the NFB experimental group no longer met the diagnostic criteria for PTSD. In the military population, as early as Peniston and Kulkosky (1991) evidenced that alpha-theta NF (8 sessions of 30 min) therapy significantly reduced anxiety-induced traumatic recurring nightmares/flashbacks in Vietnam theater veterans with combat-related PTSD and reduced the psychotropic medications. Zotev et al. (2018) showed that real-time fMRI training of amygdala reduces the PTSD severity scale, and symptoms of avoidance and hyperarousal in veterans with combat-related PTSD. The NFT results for PTSD therapy, some of which were cited above and targeted at the soldier, prompted a Comment from Young (2019) on the specific interest for soldiers. A recent study including veterans and civilians with chronic PTSD showed that an amygdala-derived-EEG-fMRI-Neurofeedback training protocol improved clinical outcomes 3 months after terminating therapy (Fruchter et al., 2024).

PTSD and TBI often coexist because brain injuries are often sustained in traumatic experiences, and are often co-morbid with chronic pain, resulting in overlapping symptoms with each of these conditions (Bryant, 2011; Moore et al., 2020). Thus, after 3 months of mobile NFT (10 min, 4 times a week) on alpha activity, chosen because consistent with pain perception pathways, veterans, suffering from both PTSD and TBI, reported a decrease in pain intensity, pain interference, depression, PTSD symptoms, anger, sleep disturbance and suicidal ideation, with no serious adverse events reported (Elbogen et al., 2021).

## 4 Considerations for the use of NFT in military personnel: physiological factors modulating NFT responsiveness

A large majority of the scientific literature reports a non-negligible proportion of subjects and/or patients (15–50%) who do not respond to the effects of NFT (non-responders) or prove unable to interact with a brain-machine interface, regardless of the protocol performed (Nan et al., 2015, 2018; Alkoby et al., 2018; Esteves et al., 2021). This inter-individual variability represents a major challenge for the development and implementation of a neurofeedback-based strategy to improve attentional skills, whether for the military or the athlete. A prerequisite for neurofeedback intervention could be to identify potential non-responders, in order to offer them personalized countermeasures to improve the effectiveness of neurofeedback. In the literature, two main psycho-physiological factors have been identified as influencing neurofeedback responsiveness: (1) the individual’s attentional capacities, dependent among other things on sleep debt, and (2) his or her level of motivation and mood to perform neurofeedback training (Kadosh and Staunton, 2019).

A third psycho-physiological factor determining the effectiveness of neurofeedback could be the quality of body awareness. Body awareness refers to an individual’s ability to perceive changes in bodily information (Mehling et al., 2009). A high level of body awareness could provide support for learning to control one’s own brain activity. Conversely, non-responders could be characterized by a low level of body awareness. To test this hypothesis, it will be interesting to study the cardiac evoked potential, an electrophysiological marker of body awareness (Coll et al., 2021), and to assess its association with the efficacy of neurofeedback training. Interestingly, the level of body awareness is modifiable by training, notably through the practice of Mindfulness meditation (Treves et al., 2019), making it possible to envisage the use of a Mindfulness intervention (e.g., a Mindfulness-Based Stress Reduction program) to improve the quality of body awareness in individuals identified as potentially non-responders. The use of Mindfulness meditation as a countermeasure to optimize the performance of neurofeedback training is supported by recent studies, which have shown its value in improving the performance of brain-machine interface applications (Jiang et al., 2021).

Finally, inter-individual variability could be explored from the angle of polymorphisms in genes of interest, known to have an influence on brain activity and alpha rhythm in particular (Bowers et al., 2015; Roy et al., 2020; Tichelman et al., 2023). The recent study of Tichelman et al. (2023) showed that variation of the adenosine A2A receptor (ADORA2A) gene is related to inter-individual variation of oscillatory alpha power recorded during rested wakefulness as well as during REM sleep.

**Figure 3:**
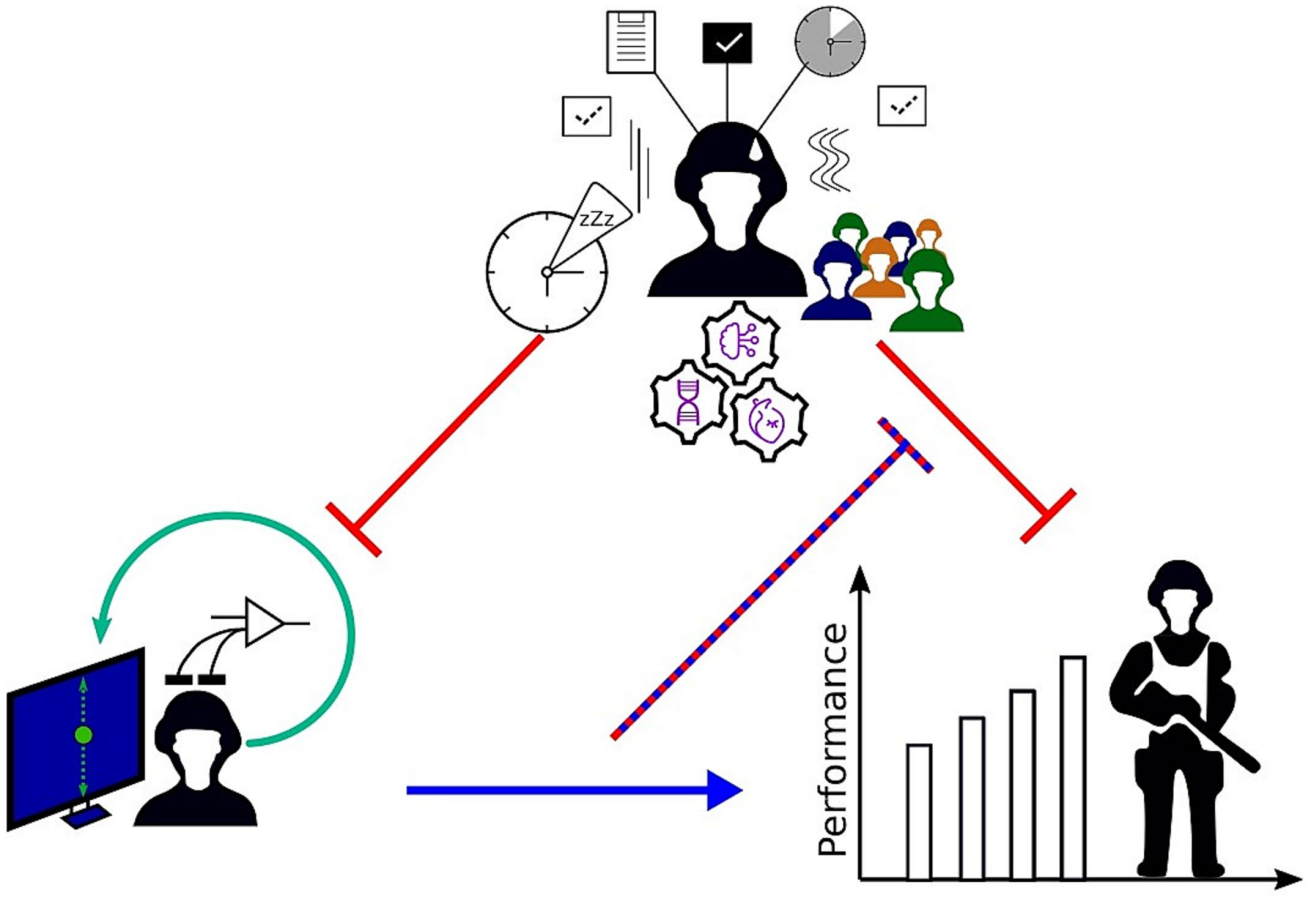
Relationships between soldier’s operational performance, NFT efficacy and intra- inter-individual variability. NFT (lower right) is expected to enhance (blue sharp arrow) operational performances (lower left) and/or to alleviate (purple dashed blunt arrow) the effects of operational constraints (upper center). At the opposite, operational constraints and intra- inter-individual variability (upper center) are known to decrease operational performances and NFT efficacy (red blunt arrows). The relationship between soldier’s operational performance, NFT efficacy and intra-individual variability. NFT (bottom left) would improve (blue pointed arrow) operational performance (bottom right) through the so-called “transfer effect” and/or alleviate (purple dashed blunt arrow) the effects of operational constraints (top center). Conversely, operational constraints and intra-individual variability (top center) are known to decrease operational performances and NFT efficacy (blunt red arrows).

In conclusion, the inter-individual variability can be at least partially explained in a multifactorial way: physiological (sleep–wake rhythm), biological (genetic polymorphisms), psychological (body awareness) and neurophysiological (characteristic features of brain activity; Figure 3). It is therefore conceivable that, depending on the professional characteristics of the soldier (pilot, sniper, etc.), the type of mission carried out (night, extreme environment, etc.) and the intrinsic properties of the individual (psychophysiology, genetics, etc.), appropriate, personalized neurofeedback training can be proposed to optimize and/or preserve the combatant’s cognitive capacities.

This inter-individual variability must therefore be taken into account (i) to determine and understand its causes (methodological, intrinsic to the individual, etc.) with a view to (ii) proposing personalized training to operational staff and (iii) developing appropriate countermeasures to enable operational staff to make effective use of the brain-machine interfaces that will be increasingly integrated into complex weapons systems.

## 5 Conclusion

To our knowledge, this review presents the interest of NFT in the military context, which involves the operational environment and its possible deleterious effects on cognitive capacity, as well as on the increased risk of brain disorders that may occur in the short/medium or even long term. As several studies have shown that EEG activity in the Alpha frequency range is correlated with attentional and memory capacities (Brickwedde et al., 2019; Bagherzadeh et al., 2020), future research should validate an Alpha-based neurofeedback training method and evaluate it cognitively in the laboratory and in the field, taking inter-individual variability into account. Furthermore, as recent data from the literature clearly underline the interest of NFT based on Alpha in particular, for the treatment of brain disorders linked to the military context, its clinical interest appears important (Young, 2019).
